# Clinical and Familial Predictors of Suicidal Ideation and Treatment Outcomes in Hospitalized Adolescents in Turkey: A Retrospective Analysis

**DOI:** 10.3390/children13050596

**Published:** 2026-04-24

**Authors:** Pınar Algedik, Azad Asaf, Şevket Duman, Mesut Yavuz

**Affiliations:** 1 Department of Psychiatry, Faculty of Medicine, Haliç University, 34060 Istanbul, Turkey; pinaralgedik@halic.edu.tr; 2 PhD Program in Statistical Information Systems, Fırat University, 23119 Elazig, Turkey; azad.asaf@saglik.gov.tr; 3 Department of Child and Adolescent Psychiatry, French Lape Hospital, 34360 Istanbul, Turkey; sevketduman@fransizlape.com; 4 Department of Child and Adolescent Psychiatry, Istanbul University-Cerrahpaşa, 34320 Istanbul, Turkey

**Keywords:** adolescents, suicidal ideation, inpatient psychiatry, major depressive disorder, length of stay, clinical improvement, suicide risk, family factors

## Abstract

**Highlights:**

**What are the main findings?**

- Major depressive disorder and a previous suicide attempt were the strongest predictors of suicidal ideation among hospitalized adolescents.
- Longer length of hospitalization was the only independent predictor of clinical improvement during inpatient treatment.

**What are the implications of the main findings?**

- Adolescents admitted with depressive disorders and prior suicide attempts should be prioritized for intensive suicide risk assessment and monitoring.
- Ensuring adequate inpatient treatment duration may improve clinical stabilization and treatment outcomes in adolescent psychiatric care.

**Abstract:**

Background/Objectives: Adolescent psychiatric inpatient units play a critical role in the management of severe psychiatric disorders and suicide risk. However, limited evidence exists regarding the clinical and familial factors that simultaneously influence suicidal ideation and treatment outcomes in hospitalized adolescents. This study aimed to identify demographic, diagnostic, and clinical predictors of suicidal ideation and clinical improvement among adolescents hospitalized in a tertiary child and adolescent psychiatry inpatient unit. Methods: This retrospective observational study included 75 adolescents aged 12–18 years who were hospitalized in a tertiary child and adolescent psychiatry inpatient unit between November 2023 and June 2025. Sociodemographic and clinical characteristics were obtained from medical records. Clinical improvement was evaluated using the Clinical Global Impression–Improvement (CGI-I) scale. Group comparisons were conducted using chi-square or Fisher’s exact tests for categorical variables and the Mann–Whitney U test for continuous variables. Multivariate logistic regression analyses were performed to determine independent predictors of suicidal ideation and clinical improvement. Results: Clinical improvement was evaluated in the full sample of adolescents (n = 75), and longer length of stay was independently associated with clinical improvement during hospitalization. Among adolescents admitted with suicidal ideation (n = 45), major depressive disorder, previous suicide attempt, irritability at admission, and fewer siblings were identified as independent predictors of suicidal ideation. In addition, female adolescents had higher rates of suicide attempts and non-suicidal self-injury, whereas psychotic disorders were more common among male adolescents. Conclusions: Suicidal ideation in hospitalized adolescents is strongly associated with affective pathology and prior suicidal behavior. Longer inpatient treatment duration appears to facilitate clinical improvement. These findings highlight the importance of early suicide risk stratification and adequate treatment duration in adolescent psychiatric inpatient care.

## 1. Introduction

Child and adolescent psychiatry inpatient units play a crucial role in the treatment of severe psychiatric disorders and the management of acute crises. Inpatient units provide a safe, structured environment for the monitoring and prevention of high-risk behaviors, such as suicidality and non-suicidal self-injury, while also serving essential functions in diagnostic clarification and improving treatment adherence [1,2,3,4,5]. In addition to crisis stabilization, inpatient units also support the delivery of effective therapeutic interventions and comprehensive biopsychosocial care that facilitate sustained recovery and continuity of care [6,7]. These multidimensional functions make inpatient psychiatric units an indispensable component of contemporary child and adolescent mental health services.

However, psychiatric hospitalization during adolescence is not without potential risks and limitations. Inpatient admission may disrupt the adolescent’s daily life, including separation from family, school, and peer relationships, which are critical for psychosocial development [8]. Prolonged or repeated hospitalizations may also contribute to unfavorable clinical trajectories, including increased risk of readmission and potential reinforcement of maladaptive patterns in certain cases [9]. Moreover, the therapeutic benefit of hospitalization may not be uniform across all patients, and outcomes may depend on factors such as continuity of care and access to timely follow-up services after discharge [10]. Therefore, psychiatric hospitalization should be considered as a complex intervention with both potential benefits and risks, requiring careful and individualized evaluation.

Indications for inpatient admission in child and adolescent psychiatry vary across countries. In European cohorts, the most common reasons for hospitalization include mood disorders, psychotic disorders, and suicide risk [11,12]. Large-scale Canadian studies have highlighted polyvictimization, family dysfunction, severe non-suicidal self-injury, and suicidal behaviors as the most influential factors for psychiatric hospitalization in adolescents [3]. Similarly, retrospective studies in Turkey have reported that the most frequent causes for inpatient admission among children and adolescents are major depressive disorder, bipolar disorder, disruptive behavior disorders, and presentations associated with suicide risk or psychotic symptoms [13,14]. These findings suggest that inpatient admissions in child and adolescent psychiatry are largely clustered around mood and behavioral disorders; however, these patterns may also be shaped by interactions between family functioning, emotional support systems, and psychosocial stressors.

Major depressive disorder, previous suicide attempts, and mood dysregulation have been identified as the strongest predictors of suicidal behavior among adolescents [15,16,17]. Other relevant factors associated with suicidal ideation include irritability, perceived social connectedness, and family functioning [18,19,20]. Length of hospitalization also plays a key role in clinical improvement, as longer inpatient stays allow for diagnostic clarification, medication optimization, and the implementation of comprehensive psychosocial interventions [21,22]. These findings highlight that adolescent psychiatric inpatient care plays a critical role in crisis management and represents a critical phase in clinical stabilization and suicide risk reduction.

Recent studies have also examined factors influencing treatment outcomes in adolescent psychiatric inpatient settings. Length of hospitalization has consistently emerged as a key factor, since extended inpatient stays facilitate clinical stabilization by providing time for diagnostic clarification, pharmacologic optimization, and the delivery of comprehensive psychosocial interventions [7,21,22]. However, this relationship may also be shaped by case complexity, as more severe or comorbid presentations often necessitate longer treatment. Other influential factors include family functioning and perceived social support [6,18,19], prior treatment history, medication adherence, and symptom acuity at admission. Rather than viewing these variables in isolation, evaluating suicidal ideation and treatment outcomes together may offer a more integrative understanding of adolescent inpatient recovery.

In summary, existing evidence highlights the clinical importance of adolescent inpatient psychiatry in both suicide risk reduction and acute clinical stabilization; however, prior research has largely examined these domains separately. Few studies have examined how demographic, diagnostic, familial, and clinical characteristics simultaneously shape both treatment response and suicidal ideation within the same inpatient setting. This limited evidence leaves an important gap in our understanding of the mechanisms underlying inpatient recovery and acute suicide risk.

The present study aims to identify the demographic, diagnostic, and clinical factors associated with clinical improvement and suicidal ideation among adolescents hospitalized in a child and adolescent psychiatry inpatient unit in Turkey. As a secondary objective, the study compares the diagnostic profiles, admission indications, length of stay, and treatment characteristics of male and female adolescents.

## 2. Materials and Methods

### 2.1. Study Design and Participants

This retrospective observational study was conducted at the child and adolescent psychiatry inpatient unit of French Lape Hospital in Istanbul, Turkey, between 13 November 2023, and 30 June 2025. The study reviewed the medical records of 75 adolescents who were hospitalized in the child and adolescent psychiatry unit and had complete diagnostic documentation based on the Diagnostic and Statistical Manual of Mental Disorders, Fifth Edition (DSM-5) criteria.

### 2.2. Clinical Setting

This inpatient unit represents one of several child and adolescent psychiatry services in Istanbul, a metropolitan city with multiple tertiary care centers. The unit has a 7-bed capacity. Despite its limited bed capacity, the unit serves a relatively broad catchment area, including referrals from different districts of the city and, in some cases, from other regions of the country. Admissions to the unit are based on clinical severity and the need for inpatient stabilization and are not limited to emergency department referrals. All patients are evaluated and diagnosed at admission by the child and adolescent psychiatrist responsible for the inpatient unit. This clinician works within a multidisciplinary treatment team consisting of one child and adolescent psychiatrist, two psychologists, one art therapist, and one physiotherapist. Therapeutic modalities include pharmacotherapy, cognitive-behavioral therapy, supportive psychotherapy, schema therapy, dialectical behavior therapy, electroconvulsive therapy (ECT), group therapy, periodic family sessions, art therapy, and structured exercise programs. Therefore, although the study is based on a single-center sample, the patient population reflects a heterogeneous clinical profile encountered in tertiary psychiatric care settings.

Patients aged between 12 and 18 years with a history of inpatient admission to the child and adolescent psychiatry unit of French Lape Hospital in Istanbul were included in the study. Patients with incomplete or insufficient data in hospitalization files or electronic medical records, those who were discharged upon family request before initiation of treatment or completion of clinical observations, day-hospital admissions performed solely for electroconvulsive therapy, and patients with neurological, metabolic, or genetic disorders were excluded from the analysis. During the study period, a total of 87 patients were hospitalized and initially screened for eligibility. Of these, 75 patients met the inclusion criteria and were included in the final analysis. A total of 12 patients were excluded for predefined reasons. Two patients were excluded due to incomplete or insufficient medical records, which were related to a technical documentation issue occurring on a specific date. In addition, four patients were excluded due to neurological, metabolic, or genetic disorders, three patients were excluded because they were admitted solely for electroconvulsive therapy (ECT), and three patients were excluded due to early discharge before completion of clinical evaluation.

## 3. Measures

### 3.1. Sociodemographic and Clinical Data Form

Participants’ sociodemographic and clinical characteristics were collected using a standardized clinical data form developed for use in child and adolescent psychiatry settings. The form collects information on age, gender, educational level, family structure, psychiatric history, previous treatments, and clinical presentation at admission. Information on previous suicide attempts was obtained from routine clinical records based on standard psychiatric assessments conducted at admission, including patient interviews, collateral information from family members, and available medical documentation. Information on household income was obtained from parental reports compiled during admission. The data were used to describe the study sample and explore potential associations between hospitalization characteristics and clinical outcomes. All sociodemographic and clinical variables were obtained from routine clinical records documented at the time of admission. These data were collected as part of standard psychiatric assessment procedures, including patient interviews, information provided by caregivers, and available medical documentation. Accordingly, all variables included in this study were systematically recorded within the clinical documentation during admission and subsequently extracted for the purposes of this retrospective analysis.

### 3.2. Clinical Global Impression Scale

The clinical global impression (CGI) scale, originally developed by Guy (1976), is a clinician-rated instrument designed to assess illness severity, global improvement, and treatment response [23]. It consists of two main components: the CGI-Severity (CGI-S) and CGI-Improvement (CGI-I) scales, both of which are rated on a 7-point Likert scale. In this inpatient unit, the CGI is routinely used as part of standard clinical practice and documented by the attending psychiatrist at both admission and discharge. In this retrospective study, CGI scores were obtained from routine clinical records using the Turkish version employed in daily clinical practice. For the purpose of analysis, clinical improvement was dichotomized based on CGI-Improvement (CGI-I) scores; scores of 1–3 were classified as “improved,” and scores of 4–7 as “not improved.” While scores of 1 and 2 indicate clinically meaningful improvement, a score of 3 reflects a limited but observable change in clinical status. In the context of short-term inpatient treatment, even such minimal improvement may represent early treatment response and clinical stabilization. Therefore, this classification was preferred to capture not only marked but also early and modest clinical improvements observed during hospitalization.

### 3.3. Indication for Hospitalization

Indication for hospitalization was obtained from clinical records and recorded in the standardized clinical data form used in the inpatient unit. Although a primary clinical reason for admission was identified by the treating clinician, patients could present with multiple concurrent indications at the time of hospitalization. Therefore, for the purposes of this study, patients could be classified into more than one indication category, and these categories were not mutually exclusive.

## 4. Statistical Analysis

Statistical analyses were performed using IBM SPSS Statistics, version 27.0 (IBM Corp., Armonk, NY, USA). Descriptive statistics were used to summarize demographic and clinical characteristics. Categorical variables were expressed as frequencies and percentages, whereas continuous variables were reported as mean ± standard deviation or median (minimum–maximum) depending on the data distribution. Group comparisons were performed using appropriate statistical tests, including chi-square and Fisher’s exact test for categorical variables and the Mann–Whitney U test for non-normally distributed continuous variables. Chi-square tests were used for categorical variables when the assumptions were met. Fisher’s exact test was applied for 2 × 2 contingency tables with small expected cell counts, whereas the Fisher–Freeman–Halton exact test was used for larger contingency tables with more than two categories. Sociodemographic and clinical variables were compared between male and female adolescents. Logistic regression models were applied to determine the independent predictors of clinical improvement and suicidal ideation. Multivariable logistic regression models were constructed by including variables that showed significant or borderline associations in univariate analyses. No prespecified covariates were included in the models. In addition, receiver operating characteristic (ROC) curve analysis was performed to evaluate the discriminative ability of length of hospitalization in predicting clinical improvement and to determine the optimal cut-off value based on sensitivity and specificity. A two-tailed *p* value < 0.05 was considered statistically significant.

## 5. Results

### 5.1. Sociodemographic Characteristics

A total of 75 patients were included in the study, comprising 23 male (30.7%) and 52 female (69.3%) adolescents. The mean age was 16.23 ± 1.50 years for male and 15.27 ± 1.56 years for female adolescents. The mean number of siblings was similar for both groups (boys 2.17 ± 1.23; girls 2.15 ± 1.07). Most of the participants were high school students (boys 69.6%; girls 84.6%) (Table 1) and lived with both parents (boys 65.2%; girls 61.5%). The rate of separated families was lower among male adolescents (boys 17.4%; girls 32.7%). Most of the participants lived with their nuclear families (boys 87.0%; girls 88.5%). The proportion of patients reporting a high household income was lower among male adolescents (boys 52.2%; girls 67.3%). The mean maternal age was 47.7 ± 3.85 years for male adolescents and 50.3 ± 5.24 years for female adolescents. Mothers were predominantly university graduates (boys 60.9%; girls 55.8%), whereas fathers mostly had high-school or university education (boys 73.9%; girls 86.5%) (Table 1).

### 5.2. Clinical Characteristics

A history of psychiatric hospitalization was lower among male adolescents (boys 30.4%; girls 42.3%). The rate of previous suicide attempts was significantly higher among female adolescents (boys 34.8%; girls 65.4%; *p* = 0.013). Non-suicidal self-injury behavior was higher among female adolescents (boys 43.5%; girls 73.1%; *p* = 0.014). Maternal chronic illness was higher among male adolescents (boys 39.1%; girls 13.5%; *p* = 0.012). The proportion of families with a psychiatric history did not differ between the two groups (boys 60.9%; girls 63.5%; *p* = 0.83) (Table 2).

### 5.3. Admission Diagnoses and Indications

The most common admission diagnoses were major depressive disorder (boys 34.8%, n = 8; girls 36.5%, n = 19; *p* = 0.883), bipolar disorder (boys 8.7%, n = 2; girls 36.5%, n = 19; *p* = 0.013), and borderline personality traits (boys 13.0%, n = 3; girls 44.2%, n = 23; *p* = 0.009). Psychotic disorders were significantly more prevalent among male adolescents (boys 34.8%; girls 1.9%; *p* < 0.001). Suicide risk was the most frequent reason for hospitalization (boys 60.9%; girls 59.6%; *p* = 0.919). Non-suicidal self-injury as the primary reason for hospitalization was significantly more common among female adolescents (boys 21.7%; girls 53.8%; *p* = 0.010). Psychotic symptoms (delusions or hallucinations) were more common among male adolescents (boys 30.4%; girls 9.6%; *p* = 0.038). The mean hospitalization period was 12.09 ± 7.73 days for male and 10.04 ± 6.34 days for female adolescents (*p* = 0.29) (Table 3).

### 5.4. Clinical Improvement and Logistic Regression Analyses

Of the total sample, 54 adolescents demonstrated clinical improvement and 11 did not, based on the CGI-Improvement (CGI-I) scale (CGI-I score 1–3 = improved). Univariate analyses were conducted for sociodemographic and clinical variables, including demographic characteristics, clinical history, admission diagnoses, admission indications, and length of hospitalization. Three variables showed significant or near-significant associations with improvement: length of hospitalization (*p* < 0.001), number of siblings (*p* = 0.015), and planned pregnancy of the mother (*p* = 0.050).

These variables were subsequently entered into the multivariate logistic regression model. The dependent variable was clinical improvement, defined as a dichotomous outcome (CGI-I score 1–3 = improved; ≥4 = not improved). In the multivariate analysis, only the length of hospitalization remained a significant independent predictor of clinical improvement (OR = 1.73; 95% CI: 1.22–2.45; *p* = 0.002), whereas number of siblings and planned pregnancy were not significant predictors. The model demonstrated strong classification performance, correctly predicting 98.4% of improved cases and 63.6% of unimproved cases, with an overall accuracy of 93.3% (Table 4).

**Figure 1:**
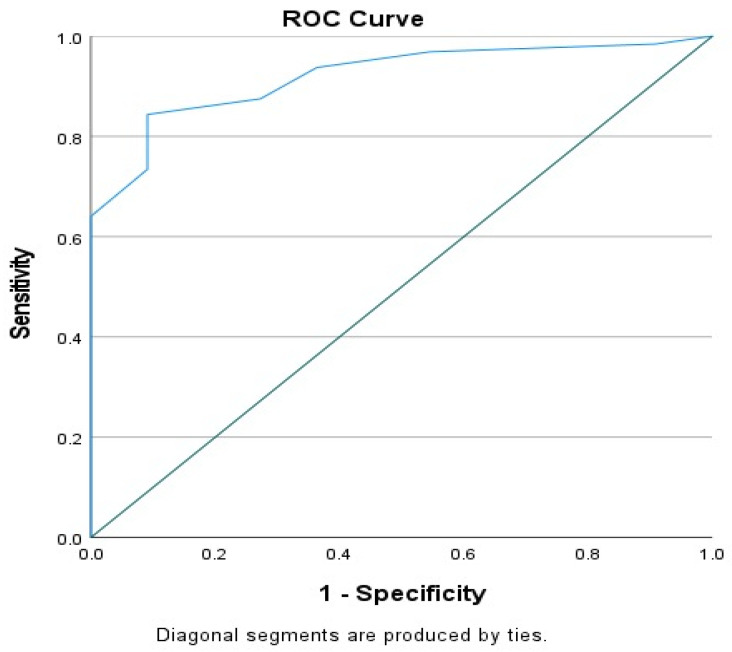
Receiver operating characteristic (ROC) curve showing the discriminative ability of length of hospitalization for predicting clinical improvement. The AUC curve is clearly stated as the ROC curve. The area under the curve (AUC) was 0.920 (95% CI: 0.851–0.988, *p* < 0.001), indicating excellent discrimination.

Receiver operating characteristic (ROC) curve analysis was performed to evaluate the discriminative ability of length of hospitalization in predicting clinical improvement. The analysis demonstrated excellent discrimination (AUC = 0.920, 95% CI: 0.851–0.988, *p* < 0.001). The optimal cut-off value was identified as 5.5 days, yielding a sensitivity of 84.4% and a specificity of 90.9% (Figure 1).

A total of 45 adolescents were admitted with suicidal ideation, while 30 were admitted for other reasons. These two groups constituted the dependent variable in the logistic regression analysis. All the sociodemographic and clinical variables considered clinically relevant (e.g., diagnostic categories, admission indications, family factors, and history of self-harm or suicide attempt) were first examined in univariate tests. Variables that showed significant or borderline associations were entered into the multivariate logistic regression model. In the final model, four factors were independently associated with suicidal ideation: having fewer siblings (OR = 0.20; *p* = 0.005), a diagnosis of major depressive disorder (OR = 18.5; *p* = 0.010), irritability as an admission indication (OR = 0.11; *p* = 0.023), and a previous suicide attempt (OR = 22.45; *p* = 0.001). The model fit was acceptable, and the regression explained a substantial proportion of the variance (−2 log likelihood = 42.10; Cox & Snell R^2^ = 0.544; Nagelkerke R^2^ = 0.735). The model correctly classified 95.6% of the adolescents with suicidal ideation and 83.3% of those without, with an overall accuracy of 90.7% (Table 5).

## 6. Discussion

This study examined the factors associated with two clinically important outcomes in hospitalized adolescents: suicidal ideation at admission and clinical improvement during inpatient treatment. In the final multivariate logistic regression model, suicidal ideation was independently associated with major depressive disorder, a history of suicide attempt, irritability at admission, and having fewer siblings. Clinical improvement was independently predicted only by a longer period of hospitalization. These results suggest that the factors that contribute to acute suicide risk differ from those that influence treatment response, highlighting the need to consider risk assessment and therapeutic planning as separate but complementary processes.

Female adolescents exhibited significantly higher rates of suicide attempt and non-suicidal self-injury, consistent with previous findings that mood disorders and internalizing symptoms are more prominent among adolescent girls [24,25,26]. The lower prevalence of such behaviors among male adolescents may be related to gender differences in help-seeking behavior and the tendency of male adolescents not to share thoughts related to non-suicidal self-injury [27].

The presence of chronic illness in mothers was more prevalent among male adolescents, suggesting that chronic maternal chronic illness may impose a greater psychological burden on boys. Some studies have shown that male adolescents tend to exhibit more externalizing and behavioral responses to family stressors, which can negatively affect their capacity for emotional regulation [28,29]. Importantly, the regression analyses showed that sex was not an independent predictor of suicidal ideation. No significant differences were found regarding a family history of psychiatric disorders or hospitalization, indicating that vulnerability to psychopathology may stem from similar familial foundations. This finding supports the view that the presence of mental illness in the family represents an important risk factor for children and adolescents, independent of sex.

Bipolar disorder and borderline personality organization were significantly more common among female adolescents, whereas psychotic and anxiety disorders were more prevalent among male adolescents, consistent with previous studies [26]. Similarly, large-scale register-based studies have demonstrated that affective and internalizing psychopathology typically emerges earlier and persists longer in female adolescents, whereas male adolescents are more likely to exhibit behavioral dysregulation, cognitive disorganization, or psychotic symptoms [30,31].

Giordano et al. [32] showed that female adolescents were more likely to present with affective instability and self-destructive behaviors, while male adolescents exhibited higher rates of psychotic and anxiety-related presentations. Consistent with this pattern, non-suicidal self-injury was markedly more common among girls, supporting the notion that emotional dysregulation and impulsivity underlie many adolescent self-injurious acts [27]. Conversely, hallucinations and delusional experiences were more frequent among male inpatients, suggesting that psychotic features may represent a gender-linked clinical phenotype in adolescent psychiatry [33].

In the present study, the logistic regression analysis conducted among adolescents admitted for suicidal ideation revealed four significant predictors: major depressive disorder, previous suicide attempt, number of siblings, and irritability. A history of major depression and previous suicidal attempts emerged as the strongest risk factors, consistent with extensive meta-analytic and longitudinal evidence identifying depressive pathology and recurrent suicidal behaviors as the most stable predictors of suicide risk [3,8,18]. While several clinical and sociodemographic variables demonstrated meaningful trends, some did not reach statistical significance, likely due to the limited sample size and the overlapping clinical characteristics of the predictors.

The study’s findings highlight that suicidal ideation in adolescents is closely associated with underlying affective and clinical variables rather than sex, as suggested in prior epidemiological reports [34,35]. One of the study’s most novel findings is the inverse association between the number of siblings and suicidal ideation. Adolescents with more siblings were less likely to be hospitalized for suicidal thoughts, suggesting that sibling relationships may act as a protective social buffer. While previous studies have linked sibling presence to reduced anxiety and depressive symptoms [36,37], direct evidence connecting sibling count with suicide risk remains limited.

From a sociological perspective, this finding aligns with Durkheim’s theory of social integration [38], which posits that stronger familial connectedness may mitigate suicide risk through enhanced belonging and emotional support. Modern empirical data similarly indicate that family cohesion and social connectedness reduce the risk of suicidal behavior among adolescents [16,18,19,39,40,41,42,43,44]. However, our data lacked qualitative measures of sibling closeness or relationship quality, which warrants further exploration in future research. Interestingly, irritability was inversely related to suicidal ideation, a pattern that diverges from most previous studies reporting irritability as a major risk factor for suicidal thoughts and behaviors in youth [20,45,46,47,48].

Several explanations may account for this discrepancy. First, irritability in some adolescents manifests as externalizing behaviors such as anger outbursts or aggression rather than internalized distress. This leads to different admission indications such as hospitalization due to aggression or behavioral dysregulation instead of suicidal ideation. Second, as the model included major depressive disorder and previous suicide attempts, the independent contribution of irritability may have been overshadowed by stronger affective predictors. Moreover, the study’s retrospective design, which relies on clinician judgment rather than self-report or ecological measures, may have influenced the directionality of this relationship. Future prospective studies can clarify these complex associations by distinguishing between episodic and chronic irritability and incorporating dimensional mood assessments. Taken together, our findings suggest that adolescent suicidal ideation arises from the interaction of affective, familial, and behavioral dimensions rather than a single diagnostic entity. The high explanatory power of the logistic model (Nagelkerke R^2^ = 0.735; overall classification accuracy = 90.7%) indicates a robust predictive pattern that emphasizes the importance of comprehensive adolescent psychiatric care that focuses on family and emotion regulation.

In the present study, multivariate logistic regression analysis identified length of hospitalization as the only significant independent predictor of clinical improvement, which was defined as a dichotomous outcome based on the CGI-I scale (CGI-I score 1–3 = improved; ≥4 = not improved). Although the number of siblings and whether the pregnancy was planned showed negative associations with recovery, these variables did not reach statistical significance, likely due to the limited sample size and asymmetric group distribution. The present study focused on group-level changes in clinical status, and individual outcomes of patients who did not show improvement in CGI scores were not analyzed separately. Therefore, the clinical course and post-discharge outcomes of these patients could not be determined from the available data. The relatively short duration of hospitalization should be interpreted in the context of intensive, multimodal inpatient care, including pharmacological treatment, structured psychotherapeutic interventions, and close clinical monitoring, which may contribute to observable short-term clinical improvement.

The finding that longer inpatient stays predict better improvement is clinically noteworthy. In addition, ROC curve analysis provided a clinically meaningful threshold for this association, identifying a cut-off value of 5.5 days with high sensitivity (84.4%) and specificity (90.9%). This finding suggests that a minimum duration of hospitalization may be required to achieve observable clinical improvement in adolescent inpatients. Prolonged hospitalization not only allows for crisis stabilization but also provides essential time for diagnostic clarification, optimization of pharmacological treatment, and effective delivery of psychosocial interventions. This result is consistent with international studies. Benarous et al. (2021) showed that longer stays in adolescent psychiatry wards facilitate comprehensive diagnostic evaluation and more effective multimodal interventions [21]. Stewart et al. (2014) demonstrated that length of stay was directly associated with recovery, particularly in patients admitted for suicide risk or severe mood disorders [22]. German multicenter studies have also found that adolescents with psychotic disorders or multiple comorbidities experience better outcomes with longer admissions [49,50]. Short-term admissions have also been found insufficient for treatment adherence in adolescents hospitalized for eating disorders, whereas longer inpatient programs showed more protective effects on both suicide risk and weight stabilization [51,52].

However, in healthcare systems with limited bed capacity, extended stays increase the clinical and administrative burden while restricting access for new admissions. The American Psychiatric Association (APA, 2022) has similarly highlighted the need to balance clinical outcomes and resource allocation [4]. Thus, while prolonged hospitalization appears to enhance clinical recovery, it should be interpreted within the framework of service capacity and equitable care delivery.

## 7. Limitations and Future Directions

The present study has some limitations. First, it was conducted in a single inpatient unit with a relatively small sample size, which may limit the generalizability of the findings. Moreover, the retrospective design allowed data collection only from existing clinical records, restricting the ability to assess longitudinal trajectories or causal relationships. In addition, information on previous suicide attempts was derived from clinical records rather than standardized prospective assessments and may therefore be subject to reporting bias or underreporting.

Number of siblings was considered a primary variable; however, the qualitative aspects of sibling relationships (e.g., emotional closeness, age gap, gender match, and biological versus step siblings) could not be examined. This limits the interpretation of sibling number as a proxy for family support. Furthermore, although CGI is a commonly used clinical measure, the reliance on a single clinician-rated scale without additional standardized assessment tools, along with the absence of interrater reliability evaluation, may have limited the objectivity and comprehensiveness of the outcome assessment. The predominance of participants from high-income households may limit the generalizability of the findings. In addition, missing data on maternal education level represents another important limitation of the study.

Future studies should adopt multicenter designs with larger and more diverse samples, allowing for a broader exploration of both clinical and familial predictors of treatment outcomes. Future studies can investigate the quantity and quality of sibling and family relationships, and include multiple informants (parents, teachers, peers) to enhance ecological validity. Prospective and longitudinal study designs can clarify the causal mechanisms underlying the associations among hospitalization duration, clinical improvement, and suicide risk.

In the current analysis, several factors appeared significant in univariate models but lost statistical significance in multivariate analysis. This pattern likely reflects the limited sample size and potential multicollinearity among the predictors. Larger-scale studies with more comprehensive statistical modeling approaches are warranted to confirm and expand these findings. Finally, the results should also be interpreted in the sociocultural context of the Turkish healthcare system, which may limit the cross-cultural generalizability of the findings.

## 8. Conclusions

In this retrospective cohort of adolescents hospitalized in a child and adolescent psychiatry unit, longer length of stay was the only independent predictor of clinical improvement, highlighting the importance of sufficient inpatient time for diagnostic clarification, pharmacologic optimization, and psychosocial interventions. Major depressive disorder and a history of non-fatal suicide attempt showed the strongest associations with suicidal ideation, whereas a higher number of siblings displayed a protective association and irritability was inversely related to suicidal ideation. Overall, the results support allocating adequate treatment duration, prioritizing risk stratification in adolescents with depressive pathology and prior attempts, and integrating family-inclusive approaches into inpatient care. Multicenter studies with dimensional assessments of mood and irritability and qualitative metrics of sibling and family relationships are needed to assess robustness and generalizability. Conducting similar analyses in outpatient adolescent populations would help to determine the clinical generalizability of these findings beyond inpatient settings.

## Figures and Tables

**Table 1:** Sociodemographic characteristics of the study participants by sex.

		Male (n = 23)	Female (n = 52)
**Age (mean ± SD)**		16.23 ± 1.50	15.27 ± 1.56
**Number of Siblings (mean ± SD)**		2.17 ± 1.23	2.15 ± 1.07
**Educational Status**	Middle School	6 (26.1%)	7 (13.5%)
	High School	16 (69.6%)	44 (84.6%)
	University	1 (4.3%)	1 (1.9%)
**Family Structure**	Both Parents	15 (65.2%)	32 (61.5%)
	Separated Parents	4 (17.4%)	17 (32.7%)
**Living Arrangement**	Nuclear Family	20 (87.0%)	46 (88.5%)
	Extended Family	2 (8.7%)	6 (11.5%)
**Household Income Level**	Low	1 (4.3%)	2 (3.8%)
	Medium	10 (43.5%)	15 (28.8%)
	High	12 (52.2%)	35 (67.3%)
**Planned Pregnancy of the Mother**	Planned	16 (69.6%)	44 (84.6%)
	Unplanned	7 (30.4%)	7 (15.3%)
**Maternal Age (mean ± SD)**		47.75 ± 3.85	50.30 ± 5.24
**Paternal Age (mean ± SD)**		49.77 ± 4.54	53.22 ± 4.83
**Maternal Education Level**	Primary School	3 (13%)	5 (9.6%)
	Middle School	2 (8.7%)	4 (7.7%)
	High School	2 (8.7%)	13 (25%)
**Paternal Education Level**	Primary School	4 (17.4%)	2 (3.8%)
	Middle School	2 (8.7%)	5 (9.6%)
	High School	6 (26.1%)	14 (26.9%)
	University	11 (47.8%)	31 (59.6%)

**Table 2:** Clinical characteristics of the study participants by sex.

		Male (n = 23)	Female (n = 52)	χ^2^ Test (df)	
**Chronic Medical Illness**	No	16 (%69.6)	37 (%71.2)	0.019 (1)	*p* = 0.889
	Yes	7 (%30.4)	15 (%28.8)		
**History of Psychiatric Hospitalization**	No	16 (%69.6)	30 (%7.7)	0.984 (1)	*p* = 0.330
	Yes	7 (%30.4)	22 (%42.3)		
**Psychotropic Medication at Admission**	No	7 (%30.4)	9 (%17.3)	1.637 (1)	*p* = 0.200
	Yes	16 (%69.6)	43 (%82.7)		
**History of Suicide Attempt**	No	15 (%65.2)	18 (%34.6)	6.061 (1)	*p* = 0.014
	Yes	8 (%34.8)	34 (%65.4)		
**History of Non-Suicidal Self-Injury**	No	13 (%58.5)	14 (%26.9)	6.064 (1)	*p* = 0.014
	Yes	10 (%43.5)	38 (%73.1)		
**Substance Use**	No	11(%47.8)	28 (%53.8)	1.058	*p* = 0.819 ^a^
	Tobacco Use	7 (%30.4)	12 (%23.1)		
	Alcohol Use	2 (%8.7)	7 (%13.5)		
	Substance Use	3 (%13.0)	5 (%9.6)		
**Family History of Psychiatric Disorder**	No	9 (%39.1)	19 (%36.5)	0.046 (1)	*p* = 0.831
	Yes	14 (%60.9)	33 (%63.5)		
**Family History of Psychiatric Hospitalization**	No	19 (%82.6)	40 (%76.9)	0.307 (1)	*p* = 0.579
	Yes	4 (%17.4)	12 (%82.1)		
**Maternal Psychiatric Disorder**	No	17 (%73.9)	30 (%57.7)	1.793 (1)	*p* = 0.181
	Yes	6 (%26.1)	22 (%42.3)		
**Maternal Chronic Medical Illness**	No	14 (%60.9)	45 (%86.5)	6.261 (1)	*p* = 0.012
	Yes	9 (%39.1)	7 (%13.5)		
**Paternal Psychiatric Disorder**	No	20 (%87.0)	42 (%80.8)	0.426 (1)	*p* = 0.514
	Yes	3 (%13.0)	10 (%19.2)		
**Paternal Chronic Medical Illness**	No	15 (%65.2)	40 (%76.9)	1.117 (1)	*p* = 0.514
	Yes	8 (%34.8)	12 (%23.1)		

^a^ Fisher-Freeman-Halton Exact Test was used.

**Table 3:** Comparison of Diagnostic and Hospitalization Characteristics by Sex.

		Male (n = 23)	Female (n = 52)	χ^2^ Test (df)	
**Admission Diagnosis**	Major Depressive Disorder	8 (34.8%)	19 (36.5%)	0.021 (1)	*p* = 0.883861
	Bipolar Disorder	2 (8.7%)	19 (36.5%)		*p* = 0.013484 ^a^
	Psychotic Disorder	8 (34.8%)	1 (1.9%)		*p* = 0.000210 ^b^
	Eating Disorder	0	3 (5.8%)		*p* = 0.548345 ^a^
	Obsessive–Compulsive Disorder	2 (8.7%)	0		*p* = 0.091171 ^a^
	Borderline personality traits	3 (13.0%)	23 (44.2%)	6.848 (1)	*p* = 0.009362 ^a^
	Conduct Disorder	3 (13.0%)	12 (23.1%)		*p* = 0.369041 ^a^
	Anxiety Disorder	3 (13.0%)	0		*p* = 0.026227 ^a^
	Other	6 (26.1%)	9 (17.3%)	0.768 (1)	*p* = 0.380779
**Comorbidity**	No	12 (52.2%)	21 (40.4%)	0.900 (1)	*p* = 0.342914
	Yes	11 (47.8%)	31 (59.6%)		
**Indication for Hospitalization**	Suicidal Ideation	14 (60.9%)	31 (59.6%)	0.100 (1)	*p* = 0.918572
	Non-Suicidal Self-Injury	5 (21.7%)	28 (53.8%)	6.672 (1)	*p* = 0.009796
	Delusions/Hallucinations	7 (30.4%)	5 (9.6%)		*p* = 0.038123 ^a^
	Behavioral Problems	2 (8.7%)	8 (15.4%)		*p* = 0.714171 ^a^
	Irritability	6 (26.1%)	17 (32.7%)	0.327 (1)	*p* = 0.567295
	Treatment Refusal	2 (8.7%)	1 (1.9%)		*p* = 0.221059 ^a^
**Multiple Admission Indications**	No	11 (47.8%)	15 (28.8%)	2.536 (1)	*p* = 0.111250
	Yes	12 (52.2%)	37 (71.2%)		
**Length of Hospitalization (days, mean ± SD)**		12.09 ± 7.728	10.04 ± 6.349		*p* = 0.286936
**Recurrent Hospitalization**	No	16 (69.6%)	30 (57.7%)	0.948 (1)	*p* = 0.330261
	Yes	7 (30.4%)	22 (42.3%)		
**Electroconvulsive Therapy Status**	No	20 (87.0%)	49 (94.2%)		*p* = 0.362983 ^a^
	Yes	3 (13.0%)	3 (5.8%)		
**Clinical Global Impression–Severity of Illness (mean ± SD)**		6.26 ± 0.864	6.35 ± 0.623		*p* = 0.949444
**Clinical Global Impression–Improvement (mean ± SD)**		2.65 ± 0.875	2.65 ± 0.714		*p* = 0.781163
**Clinical Global Impression–Side Effect Severity (mean ± SD)**		1.61 ± 0.656	1.67 ± 0.513		*p* = 0.499718

^a^ Fisher’s Exact Test was used. ^b^ Fisher-Freeman-Halton Exact Test was used.

**Table 4:** Logistic Regression Model and Classification Accuracy for Predicting Clinical Improvement.

A. Regression Coefficients					
Dependent variable: Clinical improvement (CGI-I 1–3 = Improved; CGI-I ≥4 = Not improved)					
Variable	B (Coefficient)	SE (Standard Error)	*p*-Value	OR (Exp(B))	95% CI (Lower–Upper)
**Length of Hospitalization**	0.546	0.177	0.002	1.73	1.22–2.45
**Number of Siblings**	−0.669	0.468	0.153	0.51	0.20–1.28
**Planned Pregnancy of the Mother**	−0.333	0.908	0.713	0.72	0.12–4.08
**B. Classification Accuracy**					
**Observed**	**Predicted**		**Correct (%)**		
	**Not Improved**	**Improved**			
**Not Improved**	7	4	63.6		
**Improved**	1	53	98.4		
**Overall (%)**			93.3		
Cut-off = 0.50					

**Table 5:** Logistic Regression Model, Model Fit, and Classification Accuracy for Predicting Suicidal Ideation.

A. Regression Coefficients						
Variable	B (Coefficient)	SE (Standard Error)	Wald	*p*-Value	OR (Exp(B))	95% CI (Lower–Upper)
**Number of Siblings**	−1.605	0.567	7.995	0.005	0.20	0.07–0.61
**Major Depressive Disorder**	2.918	1.130	6.664	0.010	18.50	2.02–169.48
**Irritability as Admission Indication**	−2.185	0.963	5.143	0.023	0.11	0.02–0.74
**History of Suicide Attempt**	3.111	0.959	10.523	0.001	22.45	3.43–147.06
**Conduct Disorder**	−1.760	1.119	2.476	0.116	0.17	0.02–1.54
**Constant**	2.352	1.221	3.710	0.054	10.51	—
**B. Model Fit Indices**						
**Model Fit Indices**	**Value**					
**−2 Log Likelihood**	42.100					
**Cox & Snell R^2^**	0.544					
**Nagelkerke R^2^**	0.735					
**C. Classification Accuracy**						
**Observed**	**Predicted**		**Correct (%)**			
	**Other**	**Suicidal Ideation**				
**Other (n = 30)**	25	5	83.3			
**Suicidal Ideation (n = 45)**	2	43	95.6			
**Overall (%)**	—	—	90.7			
Cut-off = 0.50						

## Data Availability

The data presented in this study are available from the corresponding author upon reasonable request. Due to privacy and ethical restrictions, the data are not publicly available.
